# Emerging role of chemokine CC motif ligand 4 related mechanisms in diabetes mellitus and cardiovascular disease: friends or foes?

**DOI:** 10.1186/s12933-016-0439-9

**Published:** 2016-08-24

**Authors:** Ting-Ting Chang, Jaw-Wen Chen

**Affiliations:** 1 Institute of Pharmacology, National Yang-Ming University, Taipei, Taiwan, R.O.C; 2 Department of Medicine, Taipei Veterans General Hospital, Taipei, Taiwan, R.O.C; 3 Cardiovascular Research Center, National Yang-Ming University, Taipei, Taiwan, R.O.C; 4 Division of Clinical Research, Department of Medical Research, Taipei Veterans General Hospital, Taipei, Taiwan, R.O.C

**Keywords:** Cardiovascular disease, Chemokine, Chemokine CC motif ligand 4, Diabetes mellitus, Fifth CC chemokine receptor

## Abstract

Chemokines are critical components in pathology. The roles of chemokine CC motif ligand 4 (CCL4) and its receptor are associated with diabetes mellitus (DM) and atherosclerosis cardiovascular diseases. However, due to the complexity of these diseases, the specific effects of CCL4 remain unclear, although recent reports have suggested that multiple pathways are related to CCL4. In this review, we provide an overview of the role and potential mechanisms of CCL4 and one of its major receptors, fifth CC chemokine receptor (CCR5), in DM and cardiovascular diseases. CCL4-related mechanisms, including CCL4 and CCR5, might provide potential therapeutic targets in DM and/or atherosclerosis cardiovascular diseases.

## Background

Chemokines and their seven-transmembrane, G-protein coupled receptors have been recognized as key mediators in the pathology of both diabetes mellitus (DM) and atherosclerosis cardiovascular diseases. These small molecular weight chemo-tactic cytokines recruit and physiologically direct cell migration. They can be broadly divided into four subfamilies, the CXC, CC, C, and CX3C families, according to their N-terminal cysteine -motifs [1]. Moreover, chemokines are also defined as “homeostatic” chemokines and “inflammatory” chemokines based on their functions. Homeostatic chemokines are constitutively secreted and involved in lymphocyte traffic, while inflammatory chemokines mediate pro-inflammatory signals and induce leukocyte recruitment to damaged tissue [2].

Diabetes patients have an increased risk of atherosclerosis cardiovascular diseases compared to people without diabetes. Diabetic vascular complications are the major cause of morbidity and early mortality in type 2 DM, suggesting the close link between type 2 DM and atherosclerosis cardiovascular disease [3–5]. Both type 2 DM and atherosclerosis cardiovascular diseases have been increasingly recognized as inflammatory related diseases. Recently, chemokine CC motif ligand 4 (CCL4), also known as macrophage inflammatory protein-1β (MIP-1β), a member of the CC chemokine family, was suggested to play a potential role in the development and/or progression of DM and atherosclerosis disease [6]. Besides, other chemokines such as chemerin may also related to inflammation and could act as novel biomarkers of acute coronary syndrome [7]. Therefore, inflammatory chemokines such as CCL4 might act as a possible node to link the presence of DM and atherosclerosis. However, the current CCL4 data are not consistent, especially for the complications of diabetes. This review focuses on the emerging evidence for the multiple roles of CCL4-related mechanisms, including those of CCL4 and one of its major receptors, fifth CC chemokine receptor (CCR5), in both experimental and clinical DM and atherosclerosis cardiovascular diseases. It may also provide the rationale for the potential role of CCL4-related mechanisms as therapeutic targets in clinical DM and atherosclerosis diseases.

### Chemokine CC motif ligand 4 (CCL4, MIP-1β)

The molecular weight of CCL4 is 7.8 kDa. Its gene is located on chromosome 17 [6]. Murine and human CCL4 proteins are synthesized as 92 amino acid precursors. Mature secreted proteins of 69 amino acids are generated by peptidases that cleave hydrophobic signal peptides [8, 9]. The three-dimensional structure of human CCL4 has been identified by heteronuclear magnetic resonance. CCL4 exists as a symmetrical homodimer. The main secondary structure elements comprise a triple-stranded antiparallel β sheet and an NH2-terminus followed by a four-residue helical turn. In general, the CCL4 dimer is elongated and cylindrical [10].

CCL4 was first isolated from the culture medium of LPS-activated macrophages [11]. LPS can impair muscle glucose uptake by both the direct effects of inflammation on myocytes, as well as by indirect NO-driven cardiovascular dysfunction [12]. CCL4 may exhibit chemoattractive ability towards different cell types, including macrophages, natural killer cells, monocytes, immature dendritic cells, and coronary endothelial cells [13–17]. In addition, CCL4 has been shown to induce calcium mobilization in natural killer cells, monocytes, leukocytes, vascular smooth muscle cells, and progenitor B cells [14–16, 18, 19]. The upregulation of circulating CCL4 levels was also observed in patients with type 2 DM and/or clinical atherosclerosis cardiovascular diseases [20–24].

### The role of CCL4 in type 1 and type 2 diabetes mellitus

Serial chemokines have been shown to be associated with the progression of both type 1 and type 2 DM. While macrophages may be implicated in the destruction of islet cells with the increase in blood glucose and the progression of both type1 and type 2 DM, CCL4 is one of the major macrophage attractants [25, 26]. While resulting in β-cell death and early islet graft loss, inflammatory stimuli with a CD40-CD40L interaction could induce the secretion of CCL4 through the Raf/MEK/ERK and NF-κB pathways in pancreatic islets [27]. CCL4 may also be produced by human islet ductal cells, which could be suppressed by sirolimus, an inhibitor of cell proliferation [28]. Clinically, the circulating CCL4 concentrations may be increased not only in the multiple islet autoantibody-positive group type 1 diabetes patients but also in prediabetic patients [20]. High-risk individuals that later developed type 1 DM had the increased secretion of the pro-inflammatory chemokine CCL4 [22]. In addition, circulating concentrations of CCL4 may be similar between type 1 and type 2 DM patients, suggesting the universal involvement of CCL4 in different stages and types of DM [29].

However, one study reported that CCL4 was significantly lower in patients with type 1 DM than in controls [30]. It was also shown that release of CCL4 from monocytes was dose-dependently induced by adiponectin [31]. Interestingly, in type 2 diabetic patients, insulin infusion could significantly suppress the expression of CCL4 in mononuclear cells. These findings are relevant because insulin is anti-inflammatory [32, 33]. Insulin also suppresses RANTES, which is atherogenic, and eotaxin, which is allergogenic [34]. Both of them bind to CCR5 as their receptor. Besides, the deletion of insulin receptor in endothelial cells leads to atherosclerosis [35]. The administration of insulin to apo E-deleted animals could also inhibit atherogenesis, suggesting the potential anti-atherosclerosis effects of insulin [36].

Taken together, in most clinical diabetes patients, circulating CCL4 levels may be increased early with the dysfunction of β-cells, even before they are extensively damaged. Further, exogenous insulin supplementation may eventually reduce CCL4 expression, likely by unloading the β-cells, as circulating CCL4 levels were inversely associated with proinsulin levels [37]. Thus, CCL4 might be produced by, rather than induce, the initial inflammatory damage on islet β-cells.

Indeed, the in vivo pathogenesis role of CCL4 in experimental diabetes may be obscured. A protective rather than aggravated role was suggested in animal studies, mainly in nonobese diabetic (NOD) mouse models. NOD mice can spontaneously develop a form of type 1 diabetes that shares some features of the human disease [38]. It was previously shown in the NOD mouse model that Th1 MNCs could enter and/or accumulate in the pancreas more rapidly than Th2 MNCs and induce immune-associated diabetes. However, both subsets induce adhesion receptors on vascular endothelium coincident with their infiltration. In vitro, Th1 cells were also distinguished from Th2 cells by the capacity to synthesize several chemokines, including lymphotactin, monocyte chemoattractant protein-1, and CCL3 (MIP-1α), whereas both subsets produced CCL4 (MIP-1β) [39]. Although the Th1/Th2 paradigm provided a conceptual framework for diseases, the opposite results of Th2-skewing agents indicate that the Th1/Th2 paradigm is too simple to explain DM pathogenesis [40–43], possibly because Th1/Th2 cytokines have pleiotropic functions beyond T helper cell phenotype modulation [44]. These studies indicate that Th1/Th2 modulation alone is insufficient for DM treatment. In addition, a recent study indicated the Treg control immune homeostasis via the CCL4/CCR5 pathway, suggesting a potential relationship between CCL4/CCR5 and Th17/Treg [45]. In another NOD mouse model, an elevated ratio of CCL3 to CCL4 in the pancreas was correlated with destructive insulitis and progression to diabetes [38]. Moreover, a decreased intra-pancreatic CCL3 to CCL4 ratio was also observed in nonobese diabetes-resistant mice. NOD.CCL3-/- mice but not NOD.CCL4-/- mice exhibited reduced destructive insulitis and were protected from type 1 DM. In NOD.*Scid* mice, which do not have functional T or B cells, CCL4 expression was undetectable in the pancreas, suggesting that the intra-pancreatic expression of CCL4 may depend on the presence of infiltrated MNCs. In NOD.*Scid* recipients, the neutralization of CCL3 with specific antibodies following transfer of diabetogenic T cells delayed the onset of diabetes, while the injection with anti-CCL4 mAb did not [46]. Furthermore, in Lewis rats, in vivo neutralization of the activity of CCL4 exacerbated the disease [47]. Blockade of IL-16 in vivo protected against type 1 DM in NOD mice by interfering with recruitment of T-cells to the pancreas, and this protection required the activity of CCL4 [48, 49]. Another study also showed that the protection from type 1 diabetes elicited by insulin-like growth factor (IGF)-I/IGF-binding protein-3 was mediated by the upregulation of CCL4 gene expression in pancreatic-draining lymph nodes, activation of the phosphatidylinositol 3-kinase and Akt/protein kinase B signaling pathway of β-cells, reduced β-cell apoptosis, and stimulation of β-cell replication [50]. It was further shown that exogenous CCL4 supplementation could suppress rather than accelerate inflammatory responses targeting islet β-cells [51]. Thus, the role of CCL4 is still undefined in DM.

Currently, almost all CCL4-related type 1 DM effects have been observed in the NOD mouse model. Ideally, more than one animal model should be investigated due to the complexity of DM [52]. Interventions in the NOD mouse studies have been reported [53]. First, the agent efficacy frequently varied when mice were treated at different ages. For example, early treatment with TNF-α exacerbated the disease but later treatment protected from disease [54]. These data suggested that the different modulations during disease progression might result in opposite effects. Second, different efficacies were observed in animal models. NOD mice and BB rats are both spontaneous autoimmune models of type 1 DM. However, nicotinamide and oral insulin showed protection in prediabetic NOD mice, but not in BB rats [55–58]. Commonly, it is easy to prevent type 1 DM onset in NOD mice if treatment is initiated early, but more difficult later in disease. As many type 1 DM patients are identified at diabetes onset, agents for DM treatment rather than prevention are urgently needed. Interestingly, previous data showed that the intra-pancreatic CCL4 concentration was relatively lower than that of CCL3 in NOD mice [50]. However, clinical data revealed that CCL4 levels were higher than CCL3 levels [21]. On the other hand, concentrations of CCL4 did not differ between groups, but CCL3 was higher in patients with latent autoimmune diabetes and type 1 diabetes than in those with type 2 diabetes and control subjects [29]. Taken together, these data imply that CCL4 levels might be different because of the different stage of DM development or the complexity of DM. As a result, although anti-CCL4 showed protective effects when the CCL4 level was relatively lower in pre-diabetic NOD mice, the effects of anti-CCL4 should also be tested when the CCL4 level is higher, as in late DM and/or in other animal models of DM.

In summary, while CCL3 contributes to the development of type 1 DM, CCL4 might play a protective role in some experimental diabetes, especially the NOD type 1 DM model. However, the role of CCL4 is much less clear in type 2 DM and might be varied in different animal models of experimental diabetes. Future studies should be required to clarify the mechanistic insights and to evaluate the clinical impact of CCL4 in the development of either type 1 or type 2 DM in humans.

### The role of CCL4 in atherosclerosis cardiovascular diseases

Previous studies showed that the inflammatory microenvironment influences cell recruitment and activation, opening new investigative fields for pathophysiological studies in cardiovascular diseases. In vitro, CCL4 was able to induce reactive oxygen species production and adhesion of THP-1 cells to human umbilical vein endothelial cells. CCL4 directly induced cell adhesion to endothelial cells through oxidative stress via PI3k–Rac1 cascades [23]. Also, macrophages under high glucose conditions released more CCL4. CCL4 secreted from macrophages under high glucose conditions is capable of inducing the endothelial expression of adhesion molecule such as E-selectin in an in vitro study [59]. Several studies have demonstrated that the E-selectin plasma level could be increased in patients with hypertension, type 2 DM, and atherosclerosis, and regard it as one of the risk factors for atherogenesis [60–63]. Furthermore, LPS-induced CCL4 production from human monocytes was significantly and positively correlated with the total and LDL cholesterol concentration [64].

In the animal model of myocardial infarction, chemokine induction in the infarct heart mediates recruitment of leukocyte subsets with distinct properties. CCL4 and its receptor CCR5 were significantly induced in the infarct mouse myocardium [65, 66]. CCL4 was also upregulated in vulnerable atherosclerosis plaques and was expressed by T cells in advanced atherosclerotic lesions in stroke patients [67, 68]. Further, elevated serum CCL4 levels have been shown to be an independent predictor of stroke and cardiovascular events in an average follow-up period of 37.2 ± 19.9 months in a cohort of hypertensive patients [23]. Patients with the highest quartile of CCL4 level showed a higher risk of stroke and cardiovascular events [23]. These data support the potential role of CCL4 in atherosclerosis disease and plaque vulnerability. However, the in vivo evidence indicating the direct contribution of CCL4 to vascular and myocardial injury is still lacking.

While similar between the non-diabetic hypertensive patients and the normotensive controls, CCL4 levels were significantly higher in the hypertensive patients with type 2 DM than in the controls. Furthermore, in these patients, CCL4 concentrations could be decreased after treatment with nifedipine, a calcium-channel blocker with antihypertensive and vascular protection effects [69]. It seems that the modulation of CCL4 in some clinical cardiovascular diseases, such as hypertension, is mainly related to the presence of type 2 DM. Although increasing evidence supports the potential role of CCL4, the mechanistic insights and clinical impact of CCL4-related atherosclerosis cardiovascular disease should be further explored.

### Chemokine receptor 5 (CCR5)

CCL4 mediates its biological effects by binding to cell surface CC chemokine receptors belonging to the G-protein-coupled receptor super family. The most well-known receptor of CCL4 is the fifth CC chemokine receptor (CCR5). CCR5 was cloned in 1996 [70] and shares 55 % amino acids identity with the first CC chemokine receptor [71]. Recent evidence has suggested that CCR5 is involved in human diseases such as infections and inflammatory diseases [72–74]. In this part, we focus on the background of CCR5 and its interactions with CCL4 in DM and cardiovascular diseases.

### The role of CCR5 in type 1 and type 2 diabetes mellitus

CCR5 may be linked with DM pathogenesis. In clinical studies, CCR5 gene polymorphisms were differentially related to the phenotypes of type 1 and type 2 DM [75, 76]. Patients with type 2 DM exhibit CCR5 over-expression on their peripheral mononuclear cells, and low-dose infusions of insulin can suppress the expression of CCR5 in mononuclear cells in obese type 2 diabetes patients [32].

In an animal model of experiment diabetes, CCR5 expression in the pancreas was associated with the development of insulitis and spontaneous type 1 DM [46]. CCR5 is also elevated in the superior cervical ganglion of type 2 diabetic rats [77]. Moreover, CCR5 is considered a novel link between obesity, adipose tissue inflammation, and insulin resistance [78, 79]. However, the data were not consistent in different animal models. In two previous studies, transient blockade of CCR5 with an anti-CCR5 mAb at 11–13 weeks of age or CCR5 deficiency significantly accelerated rather than prevented auto-immune type 1 diabetes in NOD mice [49, 80]. Furthermore, CCR5 expression at both the mRNA and protein levels was significantly increased when HUVECs were exposed to chronic high glucose [81].

Accordingly, these findings suggest the diverse roles of CCR5 in the progression of diabetes in experimental DM with different animal models. It should be further clarified if and how modulations of CCR5 could alter the development of human type 1 or type 2 DM.

### The role of CCR5 in diabetic renal disease

In clinical studies, CCR5 gene polymorphisms are not only related to the phenotypes [75, 76] but also to the risk of nephropathy in type 1 and type 2 diabetes patients [82–86]. During diabetic nephropathy, the production of chemokines, such as RANTES, CCL3, and CCL4, by glomerular and tubular cells has been reported in hyperglycemia [87–89]. It has been reported that CCR5 mRNA was upregulated in microdissected glomeruli. In clinical diabetic nephropathy, CCR5 mRNA was overexpressed in the tubulointerstitial compartment but not expressed in microalbuminuria [90]. These chemokines are able to recruit kidney monocytes by interacting with the chemokine receptor CCR5 [91]. Interestingly, the presence of CCR5∆32 is associated with better survival in type 2 diabetic patients [76].

### CCR5 in atherosclerosis cardiovascular disease

The upregulation of CCR5 may be associated with the development of atherosclerosis in type 2 diabetic patients [92]. It has been shown that the CCR5∆32 allele could be associated with decreased levels of C-reactive protein, intima-media thickness, and cardiovascular disease risk [93–95]. Reduced early onset of coronary heart disease in women is linked with CCR5 [96]. Further, the variation at the CCR5 gene was suggested to modulate the age of onset of myocardial infarction [97]. The CCR5 expression in peripheral monocytes was also increased in obese women [98]. However, another series of clinical investigations found no effects of the CCR5∆32 polymorphism on coronary artery disease or myocardial infarction in other populations [99–102]. Recently, it was shown that no chemokine receptor variant was associated with coronary artery disease, myocardial infarction or glucometabolic traits in large European ancestry cohorts [103]. Given the controversy in human studies, several investigations carried out on CCR5 in atherosclerosis mouse models, using receptor antagonism or genetic deletion, indicated that CCR5 may be important in plaque development [93, 104–108]. Interestingly, CCR5 was specifically needed for CD4 T cell homing to the atherosclerotic plaques [109].

CCR5 was discovered in smooth muscles and atherosclerotic plaques. Increased CCR5 mRNA expression was discovered in unstable carotid atherosclerotic plaques compared with the stable ones [18, 110]. An animal study revealed the association between upregulated CCR5 and disease progression in a vascular injury model using targeted nanoparticles with PET/CT imaging [111]. CCR5 was also induced in murine myocardial infarct [66]. It may mediate vasoconstriction and stimulate intimal hyperplasia in human vessels in vitro [112]. In addition, 40 % of the mononuclear cells infiltrating the infarct myocardium expressed CCR5 [65]. Furthermore, CCR5 inhibition prevents cardiac dysfunction in the SIV/Macaque Model of HIV [113]. HIV-1 binds to CCR5 as a co-receptor to enter cell macrophages and glial cells [114]. While HIV-1 infection is atherogenic [115], higher monocyte CCR5 expression and plasma IL-6 may be associated with atherosclerosis in HIV-infected individuals [116]. CCR5 antagonists could retard early ritonavir-induced atherogenesis and advanced plaque progression [117]. These findings support the involvement of CCR5, although its function is still not completely known, in the mediation of atherosclerosis and cardiovascular diseases. In 2002, orally bioavailable, synthetic, small molecule antagonists of CCR5 were tested in clinical trials. The CCR5 antagonist was shown to inhibit the proinflammatory effects of CCR5 ligands related to the pathogenesis of inflammatory diseases [118]. It was also reported that the annexin A1 fragment Ac2-26 largely diminished arterial recruitment of myeloid cells in a FPR2-dependent fashion. However, this effect was not abolished in the presence of selective antagonists to CCR5 [119]. The potential role of CCR5 in clinical atherosclerosis disease awaits further clarification.

## Conclusion

Recent findings demonstrated that both CCL4 and its receptor CCR5 play diverse roles in the inflammatory events underlying DM and cardiovascular diseases. Though it may attract macrophages to destroy islet cells, CCL4 could play a protective role in some experimental diabetes models, especially the NOD type 1 DM model. CCL4 might be the product rather than an inducer of the initial development of immune-related type 1 diabetes. However, given the specific pathological background in each individual DM disease model, the role of CCL4 should be fully investigated to ensure translation to clinical trial. On the other hand, CCL4 could also be upregulated in atherosclerosis and myocardial infarction to enhance adhesion molecule expression and accelerate the vascular inflammation response. While the findings on CCL4 neutralization may be contradictory, accumulating evidence showed the potential benefits of CCL4 blockade in experimental atherosclerosis disease but not in the development of DM. On the other hand, although the role of CCR5, a receptor of CCL4, may be diverse in different experimental DM models, experimental evidence favored the involvement of CCR5 in the progression of atherosclerosis cardiovascular disease. Accordingly, future experimental and clinical studies are worthwhile to clarify if anti-CCL4 mechanisms, including direct blocking of CCL4 and/or of CCR5, could be a promising therapeutic approach to retard the development of DM, atherosclerosis cardiovascular diseases or both.

## Abbreviations

CCL3: chemokine CC motif ligand 3
CCL4: chemokine CC motif ligand 4
CCR5: fifth CC chemokine receptor
DM: diabetes mellitus
IGF-I: insulin-like growth factor-I
MIP-1α: macrophage inflammatory protein-1α
MIP-1β: macrophage inflammatory protein-1β
NOD mice: nonobese diabetic mice
